# Supplementary material for: Diamondback Moth Larvae Trigger Host Plant Volatiles that Lure Its Adult Females for Oviposition
Source: Insects. 2020 Oct 23;11(11):725. doi: 10.3390/insects11110725 (PMC7690744; doi:10.3390/insects11110725)
Supplement: Supplementary file 1 [file insects-11-00725-s001.pdf]

insects

MDPI

Table S1. Quantities of the active synthetic compounds emitted from the cotton roll in different treatments over 12 hours.

Active Synthetic Compounds	Quantities of Chemicals Emitted from Cotton Roll (nmol) ± SE						Regression Equation
3-methylsulfinylpropyl isothiocyanate	0.01	0.50	5.00	10.00	20.00	30.00	$y = -0.001x^2 + 0.32x$
	0.071 ± 0.01	0.0651 ± 0.01	0.81 ± 0.01	3.521 ± 0.15	7.195 ± 0.22	12.26 ± 1.85	
4-methylsulfinyl-3-butenyl isothiocyanate	0.10	0.50	1.00	5.00	10.00	25.00	$y = -0.0019x^2 + 0.27x$
	0.071 ± 0.01	0.0651 ± 0.01	0.73 ± 0.01	2.301 ± 0.06	4.415 ± 0.08	7.16 ± 0.36	

Table S2. Predicted concentrations of synthetic compounds applied in the experiment.

Major Active Compounds	Quantities Detected in *Barbarea vulgaris* by GC-MS * Treatment **(μL/mL)				Predicted Concentrations of Synthetic Compounds Treatments Treatment (μL/mL)			
	**HB**	**FLB**	**TLB**	**MB**	**HB**	**FLB**	**TLB**	**MB**
3-methylsulfinylpropyl isothiocyanate	2.09	0.12	3.51	2.18	18.71	8.32	22.91	18.78
4-methylsulfinyl-3-butenyl isothiocyanate	20.13	1.56	35.5	12.01	0.10	1.36	1.89	1.43

* Data were obtained from Table 1. ** HB, healthy *B. vulgaris*; FLB, first instar larvae feeding on *B. vulgaris*; TLB, third instar larvae feeding on *B. vulgaris*; MB, mechanically damaged *B. vulgaris*.
